# Immunotherapy In Autism Spectrum Disorder; A Case Series

**DOI:** 10.1192/j.eurpsy.2023.1217

**Published:** 2023-07-19

**Authors:** S. F. Agdere, P. Topaloglu, M. Erata, G. Karacetin, Z. Yapici

**Affiliations:** 1 Department of Child and Adolescent Psychiatry, Bakirkoy Prof Dr Mazhar Osman Training and Research Hospital; 2 Division of Child Neurology, Department of Neurology, Istanbul Faculty of Medicine, Istanbul University, Istanbul, Türkiye

## Abstract

**Introduction:**

Researches has shown that a subset of the autism spectrum disorder (ASD) population presents with immune dysregulation. Based upon the immunological abnormalities, various treatment modalities have been applied to children with ASD. One immunomodulatory treatment that has been studied in ASD is intravenous immunoglobulins (IVIG).

**Objectives:**

This report is based upon two hypotheses: autism etiology may be closely related to neuroinflammation; and, an effective treatment should restore the individual’s comminication skills. In this report, we present two cases who were diagnosed with ASD and received IVIG treatment.

**Methods:**

Case A is a 5-year-old female patient who was diagnosed with ASD at the age of 3. The second case, B is a 9 -year-old male patient who is a 7th grade student and was also diagnosed with ASD at the age of 3. The first patient’s autism symptoms was noticed by her family at the age of 2. Although she had the ability to coordinate eye contact, understood simple commands, she was observed to lose all her acquired developmental skills one by one at around 18-24 months. Towards the age of 3, stereotypical movements such as turning around and flapping wings, started. Similarly, the second patient’s acquired skills were lost around at the age of 2-3, after having recurrent infections. Based on deficits in social‐emotional reciprocity, deficits in nonverbal communicative behaviors used for social interaction and restricted/repetitive patterns of behavior, both patients were diagnosed with ASD. Considering that autoimmune mechanisms may have been affected in ASD, IVIG treatment was initiated at 1 g/kg/month. Case A received IVIG once in a month, total 5 infusions had been applied. Case B received total 12 infusions with the same protocol. Treatment response was assessed with the Childhood Autism Rating Scale (CARS).

**Results:**

In these two ASD cases which were treated with IVIG ,a subjective partial reduction in autism symptoms and an objective decrease in CARS scores were detected. Current evidence suggests that there are various factors contributing to the development of autism and different combinations of these aspects give rise to different variations of some ASD subtypes. Recent studies in this field indicate a possible connection between the immune system impairments and ASD. This report, therefore, provide support for the notion that at least a subset of children with ASD might have immune abnormalities and may respond to the immune modulating effect of IVIG therapy.

**Conclusions:**

Although, autoimmune or inflammatory etiologies may not explain the majority of cases of autism, it might at least be useful to understand some. Further studies with larger number of patients should be conducted about the use of IVIG in selected patients with ASD.

**Disclosure of Interest:**

None Declared

